# Supplementary material for: Auxin apical dominance governed by the OsAsp1-OsTIF1 complex determines distinctive rice caryopses development on different branches
Source: PLoS Genet. 2020 Oct 27;16(10):e1009157. doi: 10.1371/journal.pgen.1009157 (PMC7647119; doi:10.1371/journal.pgen.1009157)
Supplement: S1 Table — (DOCX) [file pgen.1009157.s001.docx]

S1 Table Candidate genes expressed higher in 0-DAH CPB than in 5-DAH CSB and higher in 12-DAH CSB than in 5-DAH CPB.

| **RPKM**  **Gene ID** |  | **CPB** (DAH) | | | |  | **CSB** (DAH) | | | |  | **Ratio of CPB/CSB** | **Ratio of CSB/CPB** |
| --- | --- | --- | --- | --- | --- | --- | --- | --- | --- | --- | --- | --- | --- |
|  |  | 0 | 5 | 12 | 20 |  | 5 | 12 | 25 | 35 |  | 0/5 | 12/5 |
| **Os11g08200** |  | **710.52** | **18.11** | **1.95** | **2.35** |  | **146.16** | **579.42** | **8.79** | **0.34** |  | **4.86** | **31.99** |
| **Os07g39020** |  | **605.98** | **21.03** | **3.58** | **1.79** |  | **111.24** | **564.03** | **12.14** | **0.16** |  | **5.45** | **26.82** |
| **Os06g41030** |  | **200.15** | **36.29** | **7.32** | **2.22** |  | **46.29** | **224.39** | **15.27** | **2.13** |  | **4.32** | **6.18** |
| Os04g41900 |  | 57.32 | 4.32 | 0.45 | 0.28 |  | 11.91 | 33.68 | 3.10 | - |  | 4.81 | 7.80 |
| Os01g42150 |  | 28.39 | 2.19 | 3.56 | 9.59 |  | 6.16 | 13.52 | 13.63 | 15.10 |  | 4.61 | 6.16 |
| Os10g20540 |  | 23.53 | 2.45 | - | 0.33 |  | 3.73 | 13.64 | 0.72 | - |  | 6.31 | 5.57 |
| Os11g26710 |  | 20.32 | 0.85 | - | 0.17 |  | 4.77 | 25.00 | 0.37 | - |  | 4.26 | 29.41 |
| Os11g10130 |  | 19.77 | 3.69 | 2.68 | 2.35 |  | 4.42 | 18.66 | 2.11 | 0.29 |  | 4.47 | 5.06 |
| Os01g71170 |  | 17.15 | 3.95 | - | 0.08 |  | 2.46 | 16.30 | 1.50 | 0.21 |  | 6.97 | 4.13 |
| Os02g49420 |  | 14.83 | 0.05 | - | - |  | 2.84 | 4.21 | 0.04 | - |  | 5.22 | 83.04 |
| Os06g02230 |  | 14.12 | 1.06 | - | 0.09 |  | 2.74 | 5.63 | 0.27 | 0.46 |  | 5.16 | 5.31 |
| Os01g08810 |  | 10.10 | 1.14 | 1.85 | 1.57 |  | 1.91 | 5.47 | 2.97 | 2.39 |  | 5.30 | 4.81 |
| Os03g11770 |  | 8.86 | 1.38 | 0.19 | 0.70 |  | 2.07 | 7.56 | 0.96 | 2.47 |  | 4.28 | 5.49 |
| Os04g15800 |  | 7.17 | 0.73 | 0.43 | 0.37 |  | 1.49 | 4.99 | 0.41 | 0.39 |  | 4.81 | 6.87 |
| Os01g70560 |  | 7.16 | 0.98 | - | 0.27 |  | 1.34 | 4.25 | 0.59 | 0.14 |  | 5.36 | 4.32 |
| Os01g31800 |  | 7.06 | 0.34 | - | 0.63 |  | 1.59 | 2.94 | 0.30 | - |  | 4.45 | 8.65 |
| Os05g27820 |  | 6.93 | 0.46 | 0.77 | 0.71 |  | 1.39 | 5.90 | 1.55 | 0.94 |  | 5.00 | 12.83 |
| Os02g02630 |  | 4.22 | 0.19 | - | - |  | 0.71 | 5.45 | - | - |  | 5.90 | 28.54 |
| Os10g05950 |  | 4.17 | 0.06 | - | - |  | 0.81 | 1.34 | - | 0.20 |  | 5.17 | 20.76 |
| Os12g17530 |  | 3.78 | 0.56 | 0.07 | 0.04 |  | 0.90 | 2.94 | 0.23 | - |  | 4.21 | 5.29 |
| Os03g56700 |  | 3.23 | 0.08 | - | - |  | 0.41 | 1.22 | 0.15 | - |  | 7.83 | 14.70 |
| Os10g19904 |  | 3.00 | 0.27 | - | - |  | 0.68 | 2.82 | - | - |  | 4.42 | 10.38 |
| Os05g11570 |  | 2.99 | 0.05 | - | 0.06 |  | 0.69 | 2.57 | 0.22 | 0.15 |  | 4.33 | 51.03 |
| Os01g42160 |  | 2.92 | 0.49 | - | 0.20 |  | 0.61 | 2.41 | 0.72 | - |  | 4.76 | 4.90 |
| Os04g52980 |  | 2.47 | 0.42 | - | - |  | 0.52 | 2.16 | 1.10 | - |  | 4.76 | 5.19 |
| Os02g42650 |  | 2.33 | 0.12 | 0.08 | - |  | 0.57 | 1.04 | 0.04 | - |  | 4.08 | 8.36 |
| Os03g48235 |  | 2.21 | 0.52 | - | 0.24 |  | 0.41 | 2.08 | 1.15 | - |  | 5.44 | 4.00 |
| Os02g42560 |  | 1.97 | 0.03 | - | 0.04 |  | 0.48 | 2.57 | 0.45 | 0.09 |  | 4.08 | 86.50 |
| Os12g29370 |  | 1.78 | 0.07 | - | - |  | 0.35 | 1.27 | 0.12 | - |  | 5.10 | 18.16 |
| Os02g26140 |  | 1.72 | 0.41 | - | 0.12 |  | 0.32 | 2.15 | 0.18 | - |  | 5.44 | 5.30 |
| Os12g36890 |  | 1.56 | 0.24 | 0.12 | 0.02 |  | 0.38 | 1.17 | 0.16 | 0.05 |  | 4.12 | 4.81 |
| Os11g14240 |  | 1.37 | 0.18 | 0.06 | - |  | 0.29 | 1.32 | 0.18 | - |  | 4.68 | 7.50 |
| Os08g05720 |  | 1.08 | 0.34 | 1.53 | - |  | 0.21 | 2.05 | 0.45 | 1.56 |  | 5.10 | 6.05 |
| Os01g52370 |  | 1.07 | 0.21 | 0.53 | 0.13 |  | 0.26 | 1.00 | 0.70 | 0.81 |  | 4.08 | 4.76 |
