# Supplementary material for: Auxin apical dominance governed by the OsAsp1-OsTIF1 complex determines distinctive rice caryopses development on different branches
Source: PLoS Genet. 2020 Oct 27;16(10):e1009157. doi: 10.1371/journal.pgen.1009157 (PMC7647119; doi:10.1371/journal.pgen.1009157)
Supplement: S2 Table — (DOCX) [file pgen.1009157.s002.docx]

S2 Table The genotype of F1 progeny of two *asp1* independent lines self-crossed or back-crossed with WT.

| Parental Genotype (Female x Male)^a^ | Total seeds | Progeny genotypes | | | Expected ratio (*Asp1:asp1^+/--^*)^b^ | chi-squared test | P*-*value |
| --- | --- | --- | --- | --- | --- | --- | --- |
|  |  | *Asp1* | *asp1^+/-^* | *asp1^-/-^* |  |  |  |
| *asp1-1^+/-^* × *asp1-1^+/-^* | 237 | 71 | 156 | 0 | 1:2 | 0.038 | 0.848 |
| *asp1-2^+/-^ × asp1-2^+/-^* | 210 | 75 | 135 | 0 | 1:2 | 0.263 | 0.606 |
| *Asp1 × asp1-1^+/-^* | 136 | 70 | 66 | N/A | 1:1 | 0.059 | 0.808 |
| *Asp1* × *asp1-2^+/-^* | 95 | 46 | 49 | N/A | 1:1 | 0.048 | 0.827 |
| ^a^ 10 mature panicles were taken randomly from self-crossed *asp1^+/-^* plants; 6 spikelets from back-crossed *asp1^+/-^* with WT when using *asp1^+/-^* as a paternal provider. ^b^ chi-squared test was performed to evaluate the goodness-of-fit of the observed data to the predicted 1:2 ratio for self-crossed and 1:1 ratio for back-crossed plants. The genotypes of the progeny were determined by PCR. | | | | | | | |
