# Supplementary material for: Auxin apical dominance governed by the OsAsp1-OsTIF1 complex determines distinctive rice caryopses development on different branches
Source: PLoS Genet. 2020 Oct 27;16(10):e1009157. doi: 10.1371/journal.pgen.1009157 (PMC7647119; doi:10.1371/journal.pgen.1009157)
Supplement: S3 Table — (DOCX) [file pgen.1009157.s003.docx]

S3 Table The co-expression genes of *OsAsp1* from the Rice Oligo Array Database (ROAD).

| **Gene ID** | **PCC** | **RGAP Ver 6 Annotation** |
| --- | --- | --- |
| Os06g40020 | 0.6542 | DEAD-box ATP-dependent RNA helicase 52A, putative, expressed |
| Os01g55120 | 0.6511 | conserved hypothetical protein |
| Os07g39020 | 0.6472 | OsSub53 - Putative Subtilisin homologue, expressed |
| Os12g10540 | 0.6450 | OsMADS13 - MADS-box family gene with MIKCc type-box, expressed |
| Os03g11370 | 0.6442 | B3 DNA binding domain containing protein, expressed |
| Os03g53100 | 0.6332 | response regulator receiver domain containing protein, expressed |
| Os12g30520 | 0.6280 | pumilio-family RNA binding repeat containing protein, expressed |
| Os09g32948 | 0.6235 | OsMADS8 - MADS-box family gene with MIKCc type-box, expressed |
| Os05g41230 | 0.6191 | BRASSINOSTEROID INSENSITIVE 1-associated receptor kinase 1 |
| Os05g30400 | 0.6188 | expressed protein |
| **Os02g07430** | 0.6177 | **OsMADS29 - MADS-box family gene with MIKCc type-box, expressed** |
| Os05g06130 | 0.6164 | transcription factor X1, putative, expressed |
| Os06g49050 | 0.6121 | hAT dimerisation domain containing protein, expressed |
| Os08g30850 | 0.6120 | YDG/SRA domain containing protein, expressed |
| Os07g32406 | 0.6115 | expressed protein |
| Os02g41610 | 0.6110 | expressed protein |
| Os08g41950 | 0.6070 | OsMADS7 - MADS-box family gene with MIKCc type-box, expressed |
| Os02g45770 | 0.6062 | OsMADS6 - MADS-box family gene with MIKCc type-box, expressed |
| Os08g09450 | 0.6031 | OsFBL41 - F-box domain and LRR containing protein, expressed |
| Os05g07220 | 0.6028 | kelch repeat-containing protein, putative, expressed |
| Os12g06290 | 0.6025 | expressed protein |
