# Supplementary material for: Auxin apical dominance governed by the OsAsp1-OsTIF1 complex determines distinctive rice caryopses development on different branches
Source: PLoS Genet. 2020 Oct 27;16(10):e1009157. doi: 10.1371/journal.pgen.1009157 (PMC7647119; doi:10.1371/journal.pgen.1009157)
Supplement: S4 Table — (DOCX) [file pgen.1009157.s004.docx]

| S4 Table Primer and probe sequences used in the study. F, forward primer; R, reverse primer. | |
| --- | --- |
| **Gene** | **Sequence (5’-3’)** |
| **qRT-PCR Primers** |  |
| *Os11g08400* (*OsAsp1*) | **F** GCCGACTCACTCTGCACTGAC |
|  | **R** CCGAGGATGCTGTCTACCG |
| *Os08g39020* | **F** CAAGGAGAAGAACGAGGAGAAG |
|  | **R** TGACCTGATGCTTCCGATTC |
| *Os06g41030* | **F** TGTGGTGTGGATGACTGATTAC |
|  | **R** CCACCGGTTTCCTCGTTTAT |
| *OsCyCB1* | **F** GCTGCCTCTGCTGTCTATG |
|  | **R** CCAATAGCTGTGACTCGGTAAA |
| *OsKPR1* | **F** GCGAGAGGAGAGAAACAACTC |
|  | **R** TTGCCGCAGAAGGTAGTG |
| *OsCCS52A* | **F** CCGTGCCCAGGATGATTATATTAG |
|  | **R** AGATGCAAGCTGACGGTTATC |
| *OsTAA1* | **F** GTCCTGCCAAATAGGTTCTCA |
|  | **R** TCACGCAGGCACTGATCTAC |
| *OsYUCCA1* | **F** TCATCGGACGCCCTCAACGTCGC |
|  | **R** GGCAGAGCAAGATTATCAGTC |
| *OsTIF1* | **F** GAGAAAGACAAGGATGGCAAGA |
|  | **R** GAGGAAGAAGACGAGGAGTAGA |
| *OsTIF2* | **F** GGCGAAACATCGTGGAGATAA |
|  | **R** CGTTGTGCACCTTGAGGAT |
| *OsActin1* | **F** GGAGCGTGGTTACTCATTCA |
|  | **R** GTCTCCATTTCCTGGTCATAGTC |
| *AtTAA1* | **F** GACATATGTTCGGTCGGGTATG  **R** GATGGTTCCGTCAGGGTTATTAG |
| *AtYUCCA8* | **F** GATTACAAGTCCGGCGAGAA  **R** CATGGAAGGCTTAGCGAAATG |
| *AtYUCCA9* | **F** CAGACGGAGAGGAGATGGAATA  **R** CTCCGTCAAACTCCGTCATAAG |
| *AtUBQ10* | **F** CACACTCCACTTGGTCTTGCGT  **R** TGGTCTTTCCGGTGAGAGTCTTCA |
| **ChIP-qPCR Primers** |  |
| 1 *P_OsAsp1_* -1412 | **F** TCACCACTATAGAGATCAGT |
|  | **R** ATTTTGCTCATTTGGTATAG |
| 2 *P_OsAsp1_* -1217 | **F** TGTGTTGTGATAGCTCATAA |
|  | **R** TTGTTATTAAAAGGATAATA |
| 3 *P_OsAsp1_* -770 | **F** CTTTTCTGAAGTTGACAAAT |
|  | **R** TCTATAGACTAGTCAGCATT |
| 4 *P_OsAsp1_* NC (GUS) | **F** GAATACGGCGTGGATACGTTAG |
|  | **R** GATCAAAGACGCGGTGATACA |
| 5 *P_OsTAA1_* -2349 | **F** GAGTGTGTAGTAGACAAACCAAATG |
|  | **R** GCATCGATGATTAGTAGGAGCTTA |
| 6 *P_OsTAA1_* -1748 | **F** CGGCTGCGTATTGAGTTACA |
|  | **R** CTGCCGTACAAGGAGTTGATAG |
| 7 *P_OsTAA1_* -377 | **F** GCCTGTAGTACACTAGTACCTCT |
|  | **R** CTCAAGCTGACGAGTGATGAA |
|  |  |
| **Semi RT-PCR Primers** |  |
| Semi-OsAsp1 | **F** CCTCCATCACACCATGACTG |
|  | **R** TGCTGAGAGGGAGAACCTGT |
| Semi-MADS29 | **F** GAGGGAACTCATCGAGCATTAC |
|  | **R** CAGGAAGCTGTTCTGGTCTTC |
| Semi-OsActin1 | **F** TCCTCCGTGGAGAAGAGCTA |
|  | **R** GCGATAACAGCTCCTCTTGG |
| Semi-OsEF | **F** GCCAAAGGAAGCCAAGAAAG |
|  | **R** AAAGCCTCGCCCTCAAAT |
| **Genotyping Primers** |  |
| *asp1-1* LB | **F** TGGAAACGGACACTTTCTCC |
| *asp1-1* RB | **R** AACGTTGATGGGTTCTCTGG |
| *asp1-2* LB | **F**  AGCACATGTTTGATCGTTCG |
| *asp1-2* RB | **R**  TCAGTGCGATAGAATTCCCC |
| LBB | GTTACGTCCTGTAGAAACCCCAA |
| **Cloning and Vecter Constructing Primers** | |
| **35S::OsAsp1-GFP** |  |
| OsAsp1 -Full | **F** gatctgACTGCAAGGTTGGCACTGCTC |
|  | **R** ctagtGAGACGCGAAGTGATCGCAGA |
| **2*35S::MADS29-GFP** |  |
| MADS-Full | **F** ggatccATGGGGCGCGGCAAG |
|  | **R** gcggccgcCCACAGCTGCAGGCCGT |
| **Ubi::TIF2-HA** |  |
| TIF2-FULL | **F** aagcttCAATGGAAGCTAGTGCAGCCAAG |
|  | **R** actagtGTAGCCACTGTATATGACAACAAA |
| **pET32a MADS29** |  |
| MADS29-Full | **F** ggatccATGGGGCGCGGCAAG |
|  | **R** aagcttCCACAGCTGCAGGCCGT |
| **pET32a TIF1** |  |
| OsTIF1-Full | **F** gaattcATGGAAGCTAGTGCAGCCAAG |
|  | **R** aagcttGTAGCCACTGTATATGACAACAAA |
| **AD/BD-OsAsp1** |  |
| OsAsp1 -Full | **F** catatgACTGCAAGGTTGGCACTGCT |
|  | **R** cccgggTCAGAGACGCGAAGTGATCG |
| **AD/BD-OsTIF1** |  |
| OsTIF1 -Full | **F** catatgGAAGCTAGTGCAGCCAAGGAG |
|  | **R** gaattcCTAGTAGCCACTGTATATGA |
| **P_OsAsp1_:GUS** |  |
| P_OsAsp1_ | **F** atcgatCTCTTCTTCGCTCAACTAAACCC |
|  | **R** gtcgacGGTGTGATGGAGGTGGAGCT |
| **P_OsTAA1_:GUS** |  |
| pOsTAA1 | **F** aagcttAAAATGGAGAGAATGGAAGGA |
|  | **R** gtcgacCTGGATTACAATAAAGAAAACGA |
| **EMSA Probe Primers for *P_OsAsp1_* (product 60 bp)** | |
| Site |  |
| -1412 | **F** TCACCACTATAGAGATCAGT |
|  | **R** ATTTTGCTCATTTGGTATAG |
| -1217 | **F** TGTGTTGTGATAGCTCATAA |
|  | **R** TTGTTATTAAAAGGATAATA |
| -770 | **F** CTTTTCTGAAGTTGACAAAT |
|  | **R** TCTATAGACTAGTCAGCATT |
| **EMSA Probe Primer for *P_OsTAA1_* (product 30 bp)** | |
| Site -2349 | **F** tcctactaatcatcgatgcttcatcaaagc |
|  | **R** gctttgatgaagcatcgatgattagtagga |
| Site-1748 | **F** acttggatttcatcactagtatgcgtcgag |
|  | **R** ctcgacgcatactagtgatgaaatccaagt |
| Site-377 | **F** gtgaagcctgtagtacactagtacctctgt |
|  | **R** acagaggtactagtgtactacaggcttcac |
