# Supplementary figures and images for: Auxin apical dominance governed by the OsAsp1-OsTIF1 complex determines distinctive rice caryopses development on different branches
Source: PLoS Genet. 2020 Oct 27;16(10):e1009157. doi: 10.1371/journal.pgen.1009157 (PMC7647119; doi:10.1371/journal.pgen.1009157)

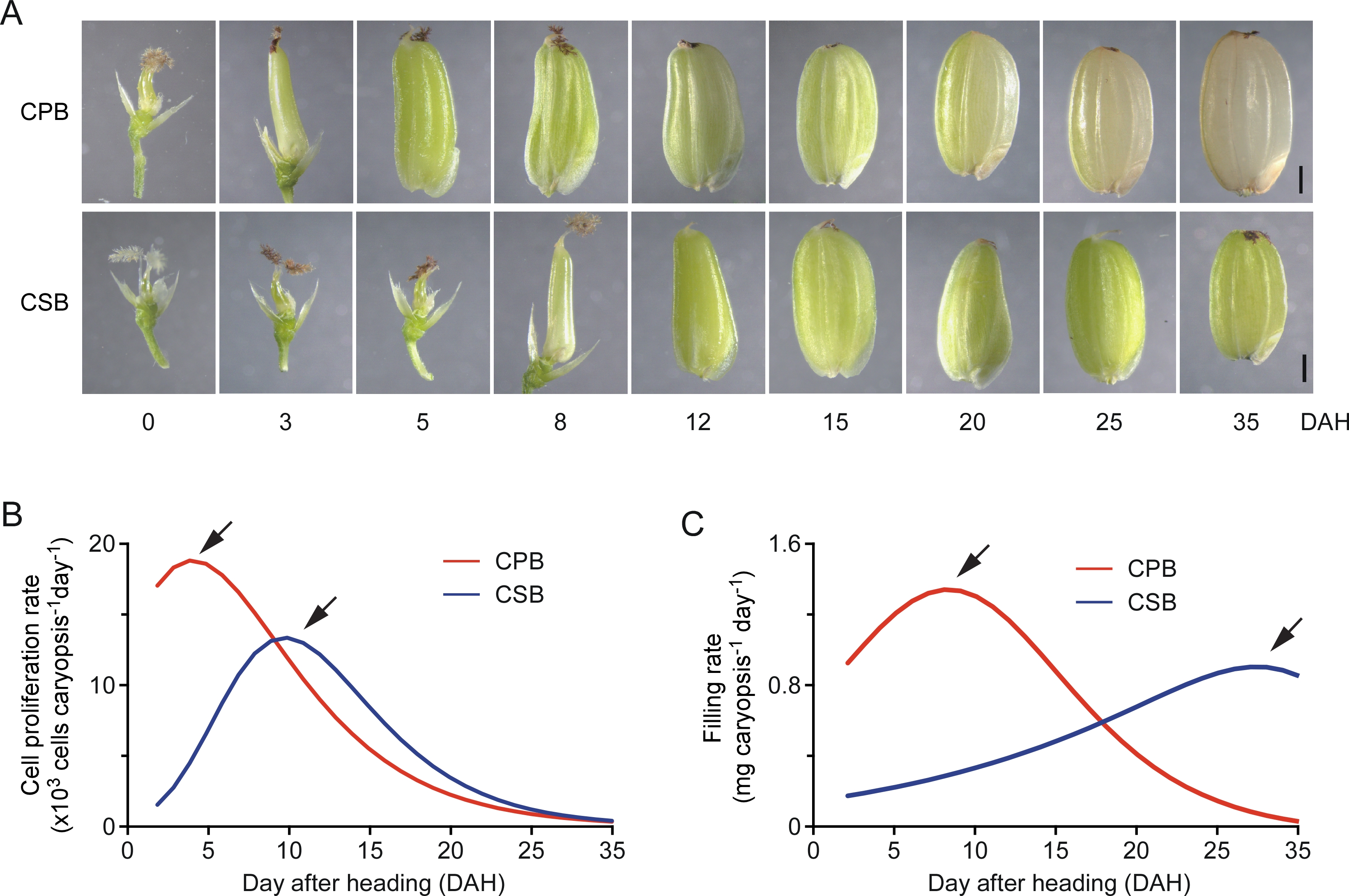

Supplement: S1 Fig — A, Developmental phenotypes of CPBs and CSBs during the period after heading. Scale bars, 20 μm. B, Cell proliferation rates of CPBs and CSBs during the period after heading. C, Filling rates of CPBs and CSBs during the period after heading. Arrows indicate the highest filling rates. Red and blue lines indicate CPBs and CSBs, respectively. (JPG) [file pgen.1009157.s005.jpg]

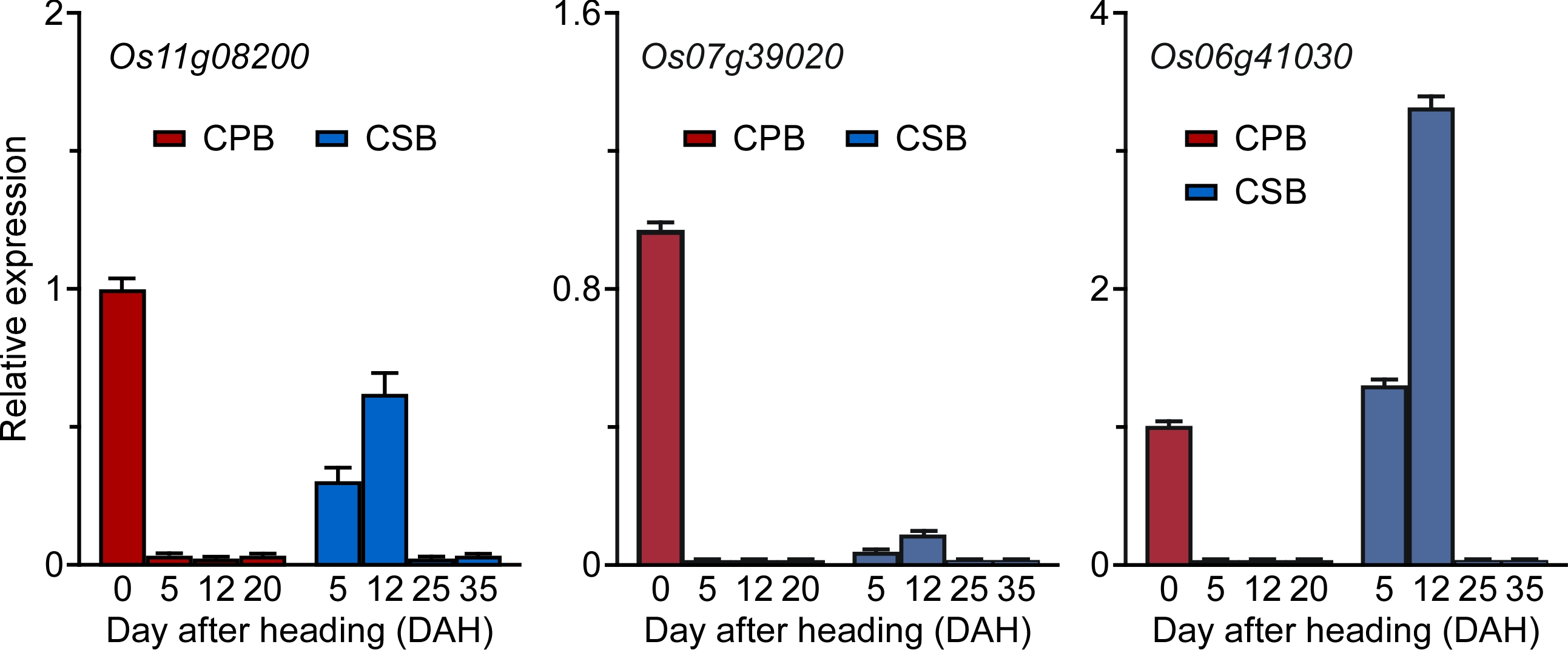

Supplement: S2 Fig — RNA samples were extracted from caryopses at eight development stages (CPB-0, -5, -12, -20 and CSB-5, -12, -25, -35). The expression of each target gene was normalised to that of OsActin1, and the relative expression of CPB-0 was set to 1.0. Data are means ± standard deviation (SD) of three biological replicates. (JPG) [file pgen.1009157.s006.jpg]

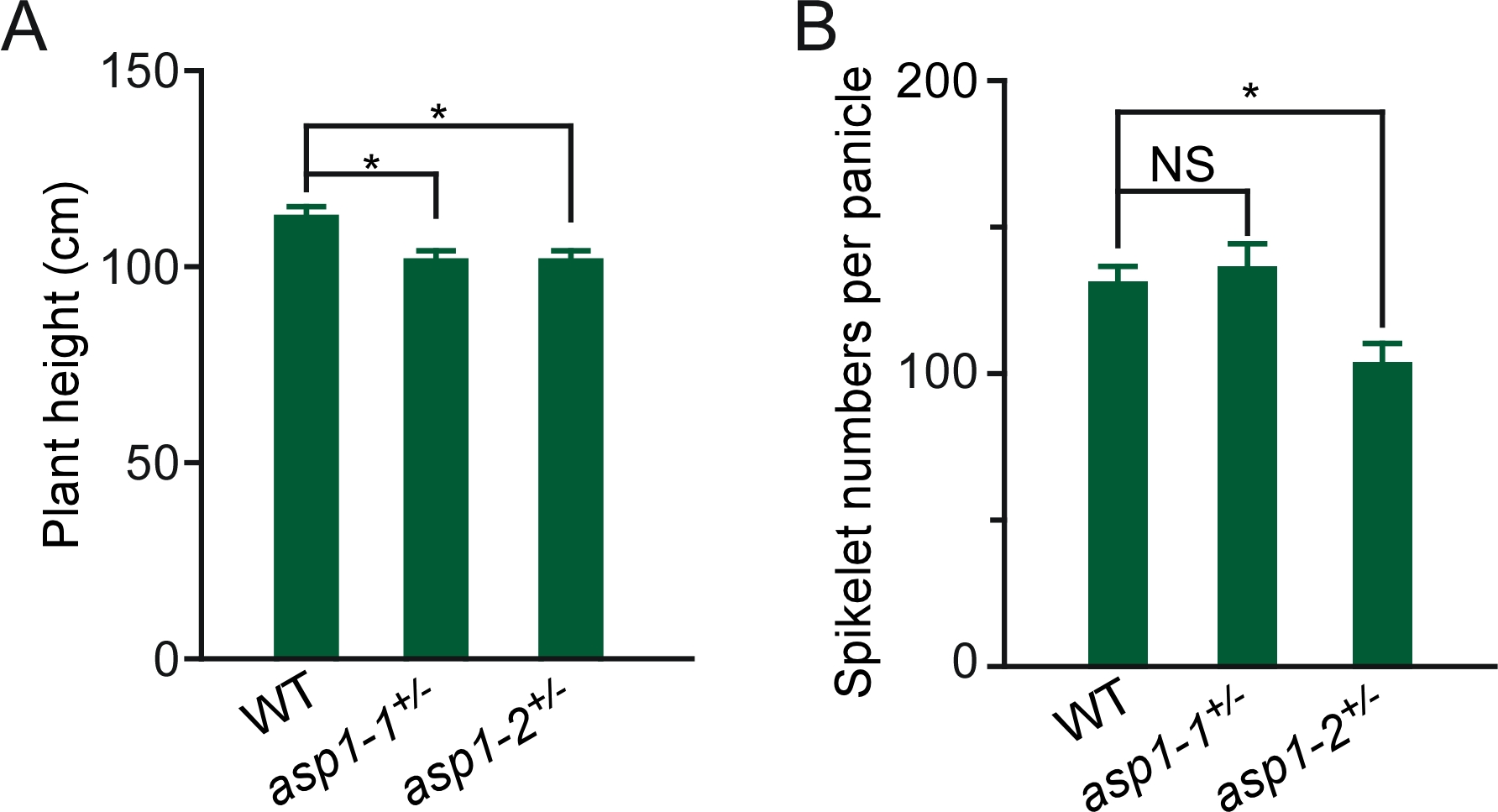

Supplement: S3 Fig — A, Plant height of rice wild-type (WT) and mutant lines asp1-1+/–and asp1-2+/–at 40 DAH. B, Spikelet numbers per panicle in the WT, asp1-1+/–, and asp1-2+/–. Values are means ± SD (n = 10). *0.01 < P < 0.5 (Student’s t-test). NS, no significant difference. (JPG) [file pgen.1009157.s007.jpg]

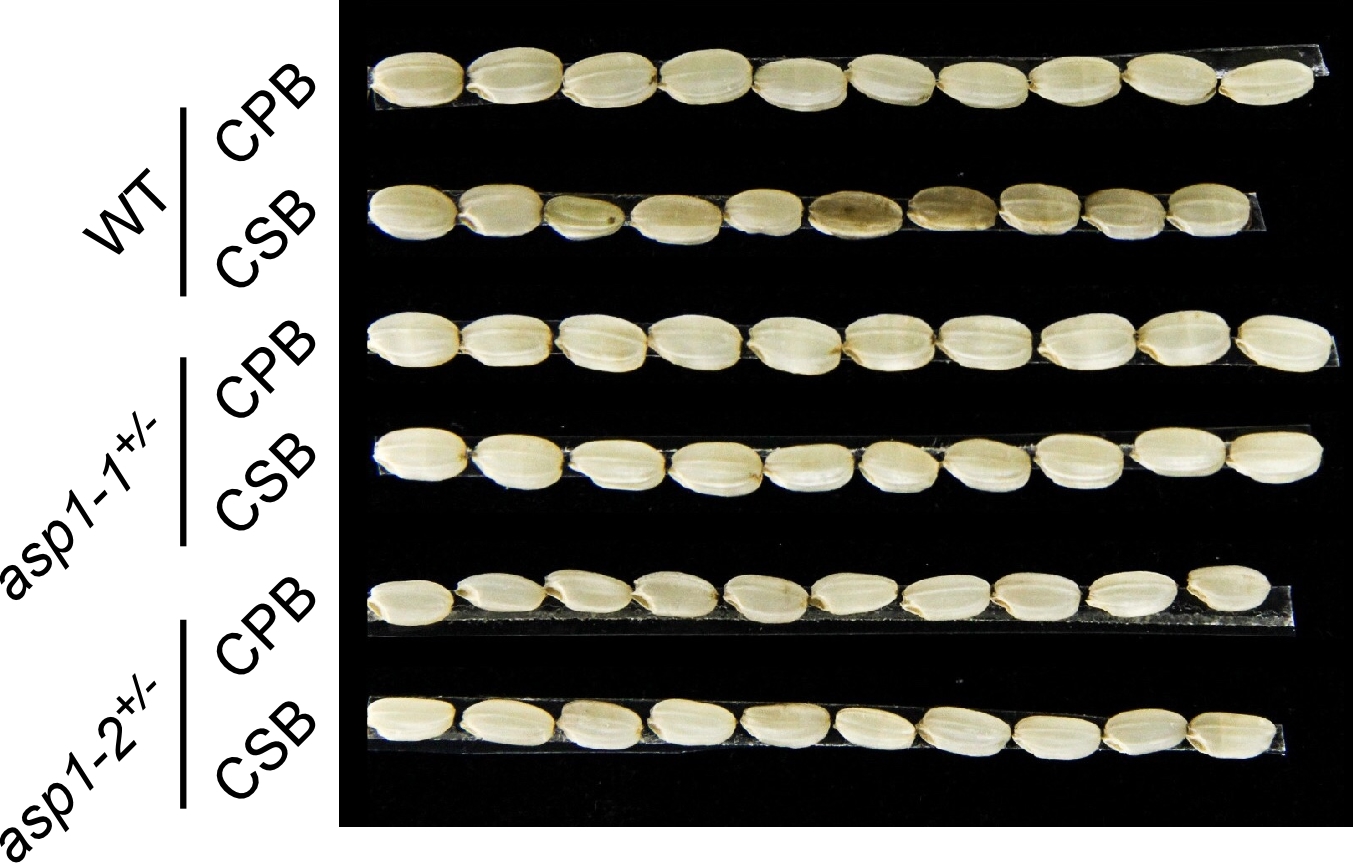

Supplement: S4 Fig — The developed grains were collected at 40 DAH from CPBs and CSBs in all plants. (JPG) [file pgen.1009157.s008.jpg]

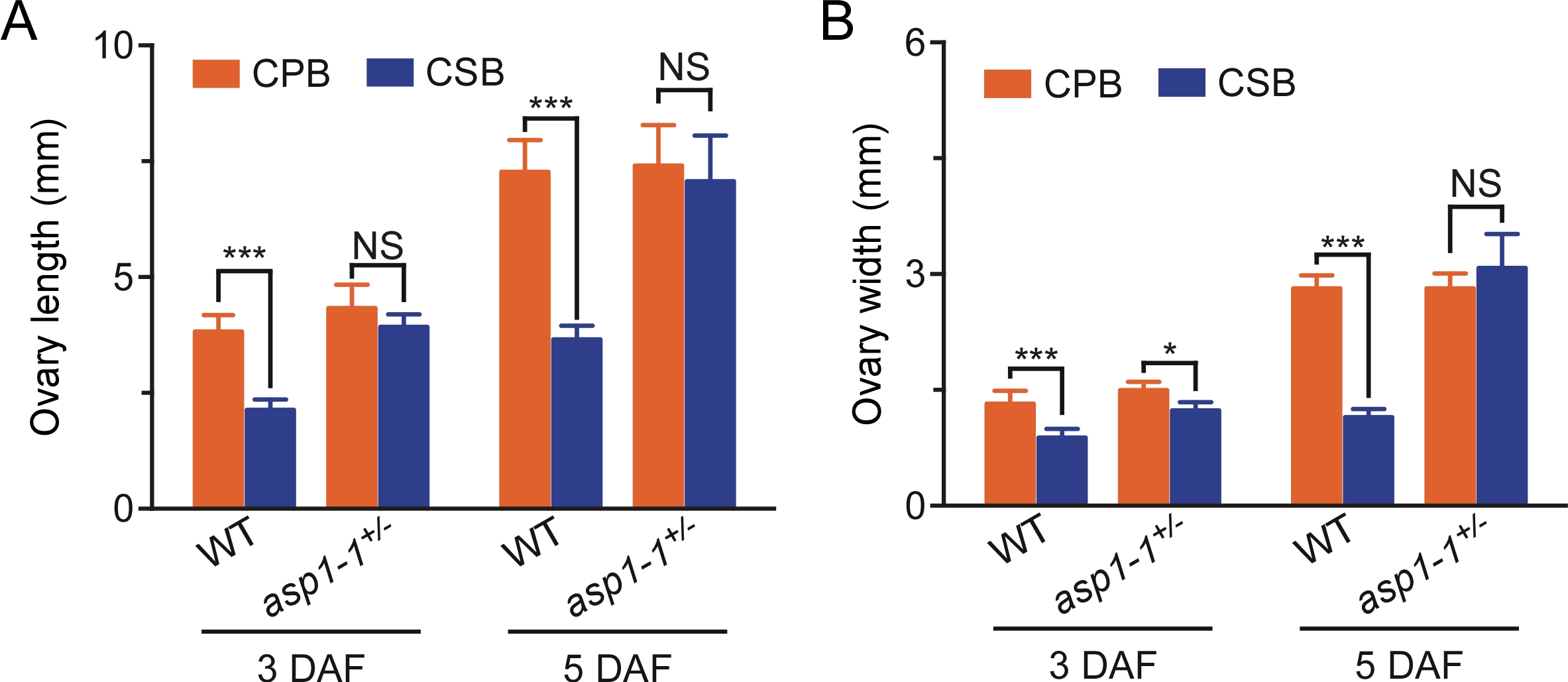

Supplement: S5 Fig — A, Ovary length. B, Ovary width. Values are means ± SD (n = 6). NS, no significant difference; *0.01 < P < 0.5; ***P < 0.001 (Student’s t-test). (JPG) [file pgen.1009157.s009.jpg]

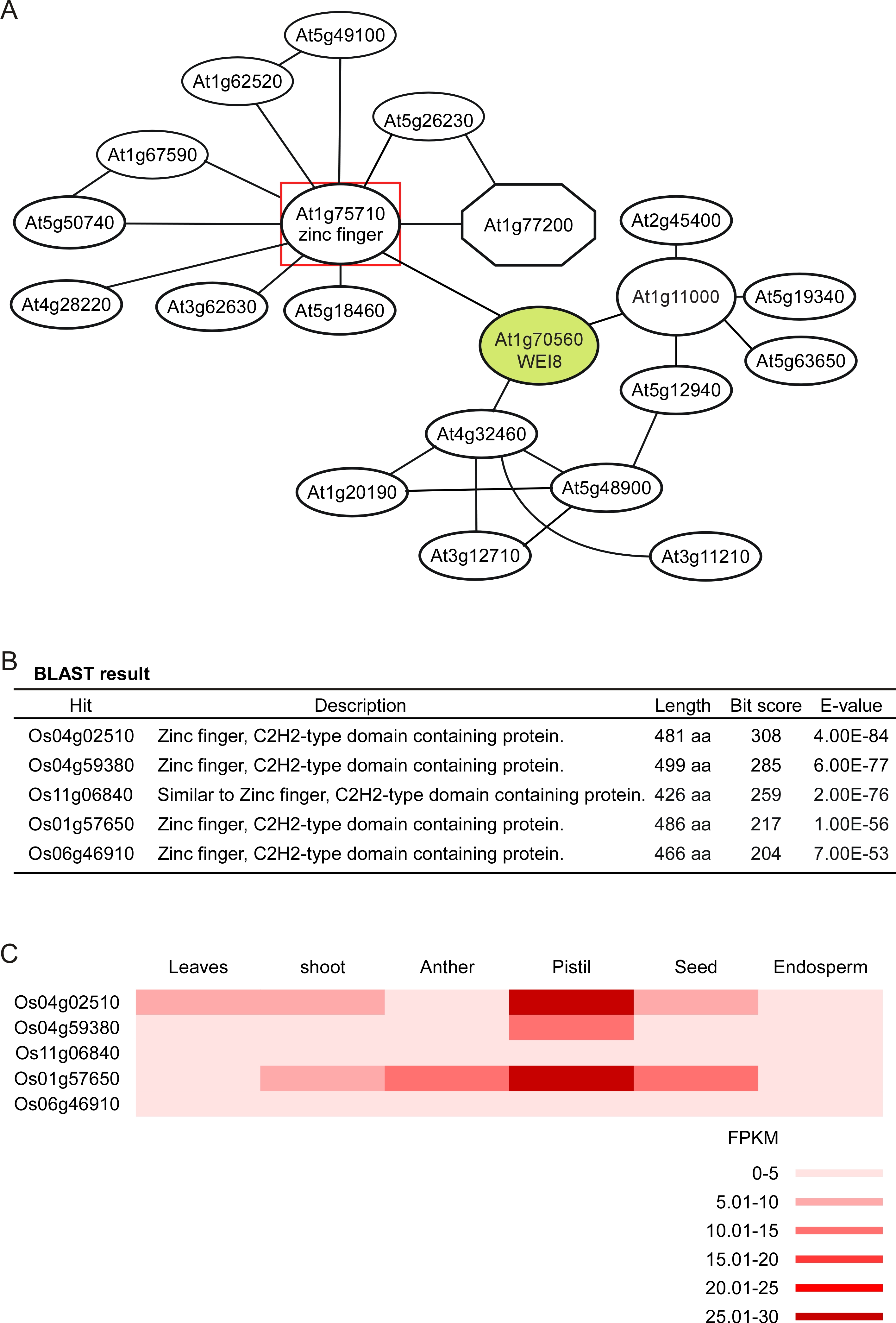

Supplement: S6 Fig — A, Genes co-expressed with AtTAA1 (At1g70560, WEI8) were forecast from an Arabidopsis gene co-expression network (http://atted.jp/). Red rectangle indicates the potential transcription factor (At1g75710, a zinc-finger protein) of AtTAA1. B, Genes homologous to At1g75710 were obtained by BLAST searching of the rice genome sequence at http://rapdb.dna.affrc.go.jp/. C, Expression profiles of five rice homologous genes of At1g75710 in different tissues and organs. The RNA sequencing (RNA-seq) expression values of these five genes (in fragments per kb per million mapped reads, FPKM) were extracted from http://rice.plantbiology.msu.edu/index.shtml. Gene expression values are presented as a heat map. (JPG) [file pgen.1009157.s010.jpg]

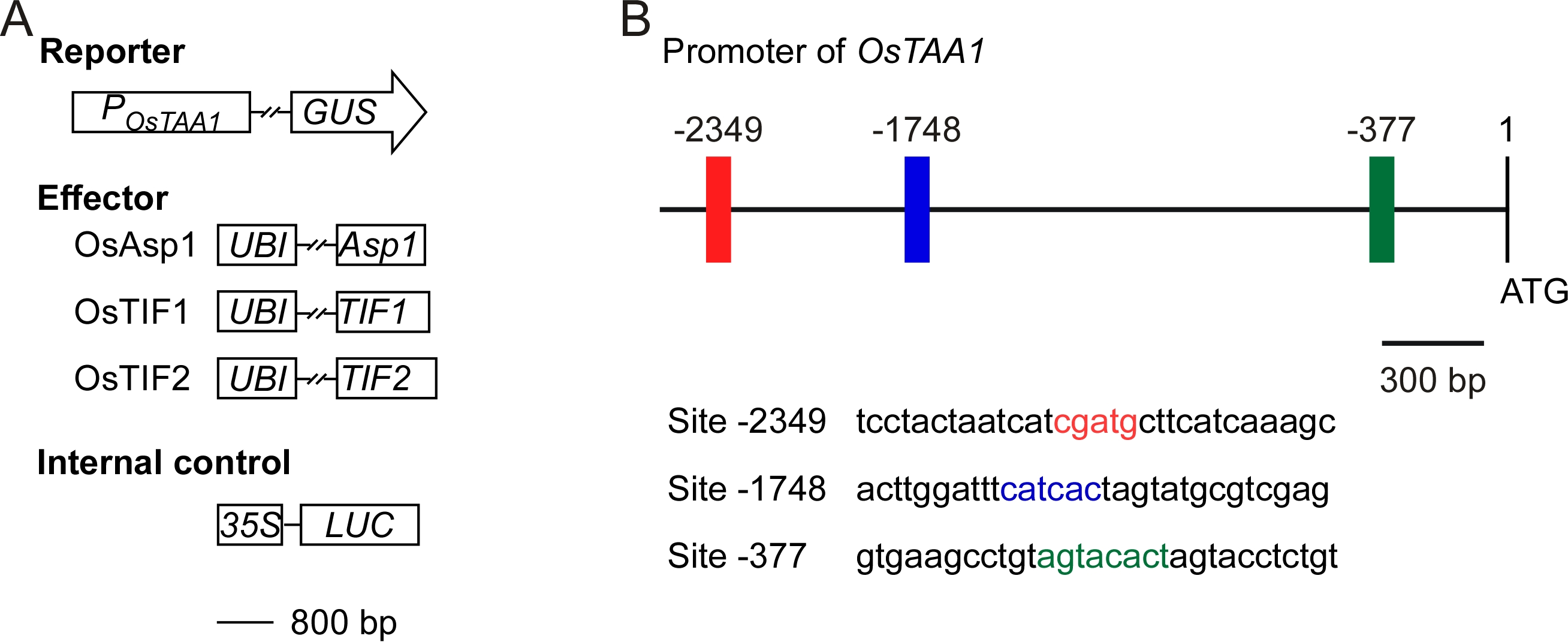

Supplement: S7 Fig — A, Constructs used for GUS assays. LUC, firefly luciferase; GUS, beta-glucuronidase. Scale bars, 800 bp. B, OsTIF1 binding sites located in the 2500 bp upstream from the ATG site of OsTAA1. Red, blue, and green boxes indicate the OsTIF1 binding sites. The 30-bp sequence around the binding site was used as a probe for the electrophoretic mobility shift (EMSA) assay (Fig 2E). Scale bars, 300 bp. (JPG) [file pgen.1009157.s011.jpg]

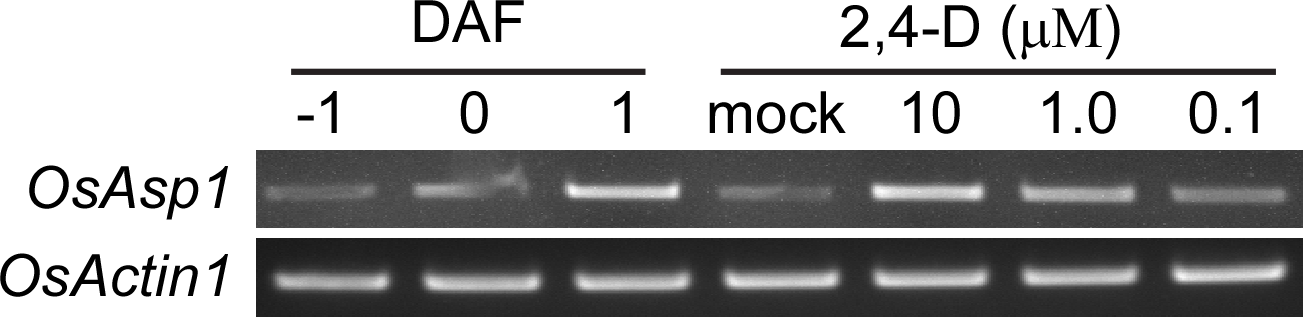

Supplement: S8 Fig — (TIF) [file pgen.1009157.s012.tif]

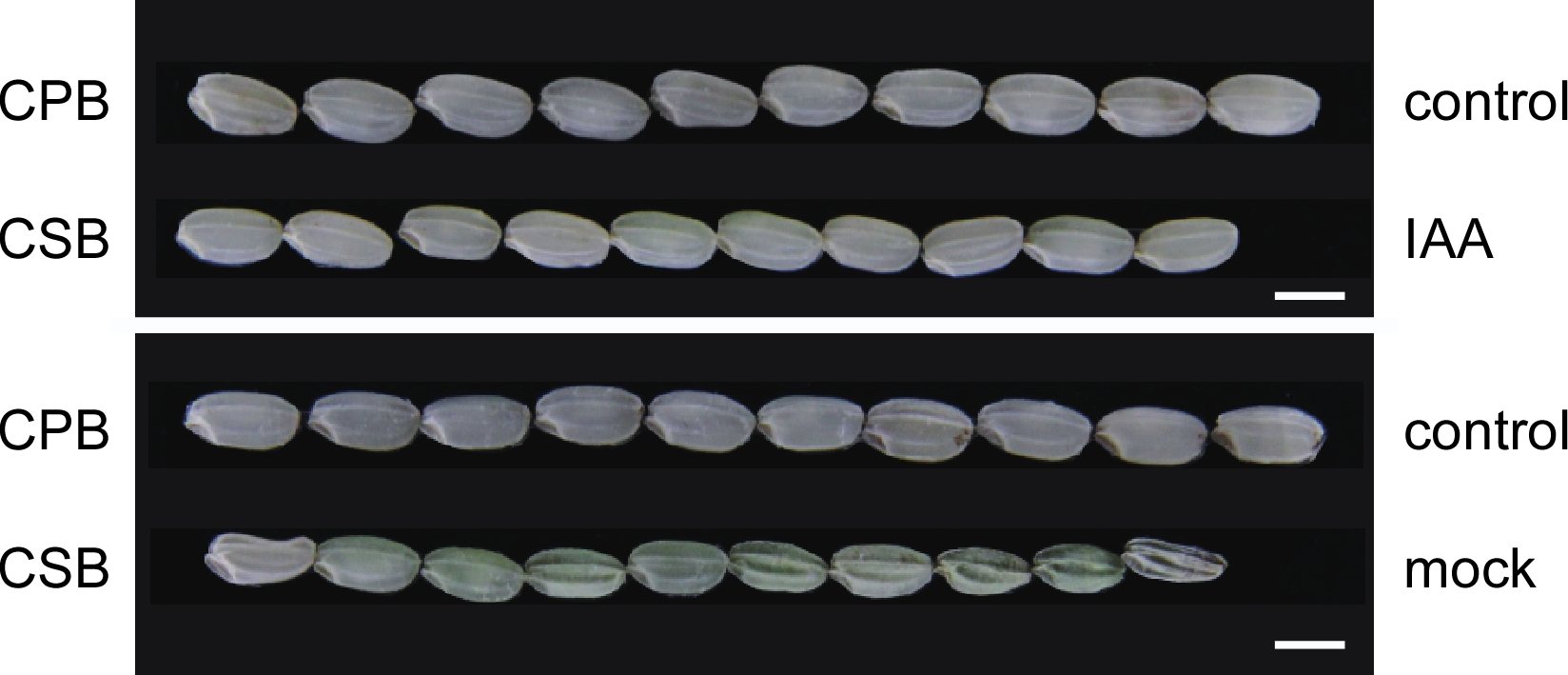

Supplement: S9 Fig — CPBs without treatment were used as a control. Scale bars, 10 mm. (JPG) [file pgen.1009157.s013.jpg]

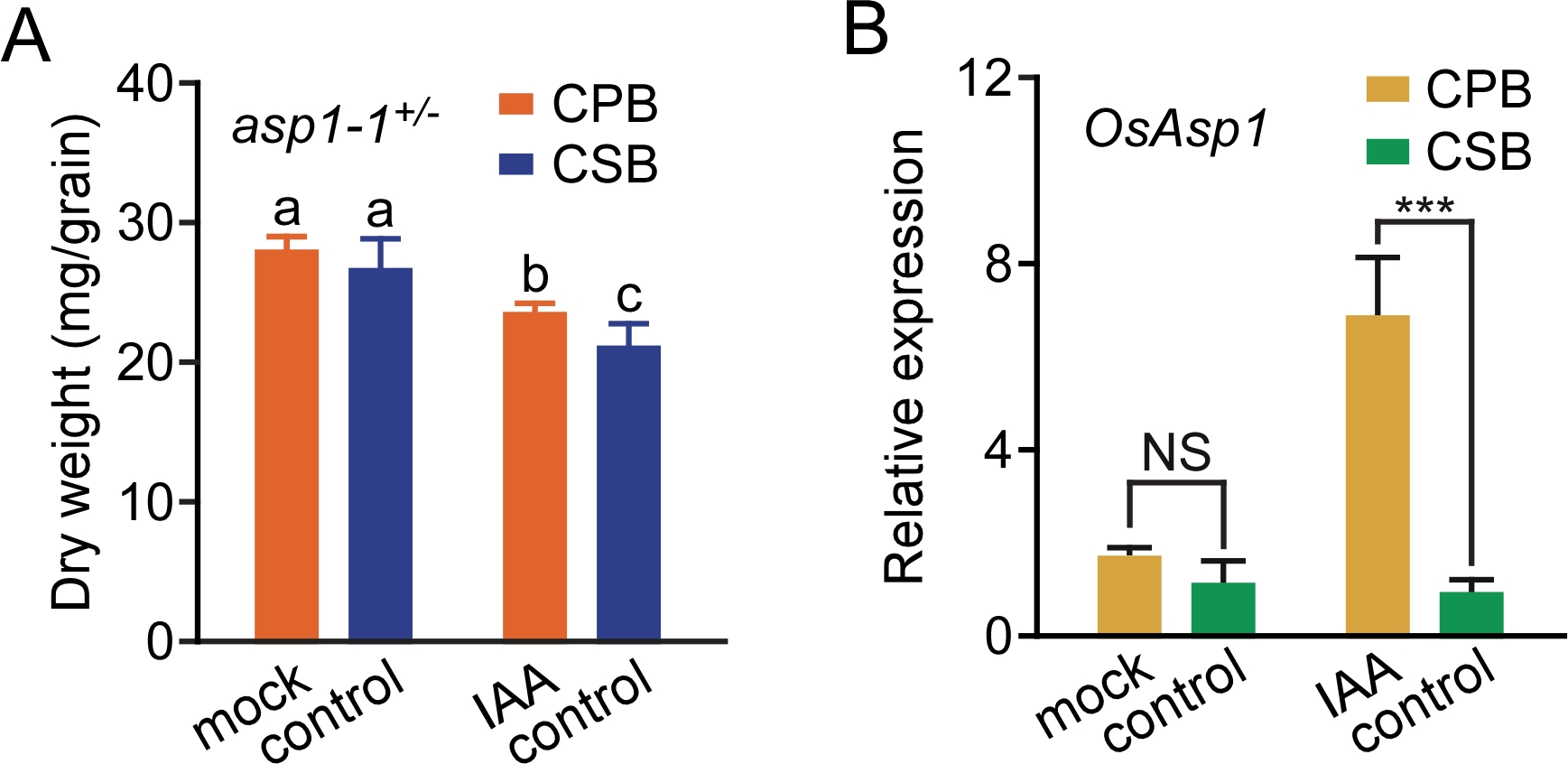

Supplement: S10 Fig — A, Mature grain dry weight of CPBs and CSBs for asp1-1+/–with IAA treatment. CPBs were treated with IAA or methanol as a control (mock). CSBs without treatment were used as a control check. Values are means ± SD (n = 30). Different letters indicate significant differences at P < 0.05 (Student’s t-test). B, Transcription levels of OsAsp1 were detected by qRT-PCR in CPBs and CSBs in asp1-1+/–with the same IAA treatment as (A) at 3 DAF. OsAsp1 expression was normalised to that of OsActin1, and the relative expression of CSBs was set at 1.0. Values are means ± SD of three independent experiments. NS, no significant difference; ***P < 0.001 (Student’s t-test). (JPG) [file pgen.1009157.s014.jpg]

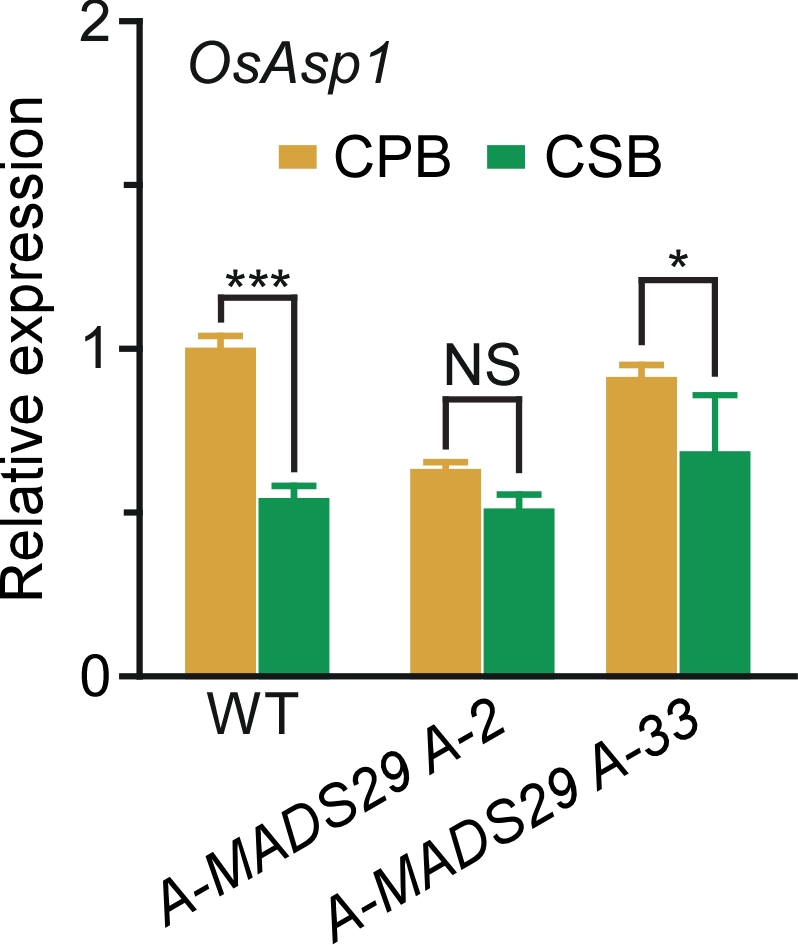

Supplement: S11 Fig — The expression of OsAsp1 was normalised to that of OsActin1, and relative expression in CPBs in the WT was set at 1.0. Values are means ± SD of three independent experiments. NS, no significant difference; *0.01 < P < 0.5; ***P < 0.001 (Student’s t-test). (JPG) [file pgen.1009157.s015.jpg]

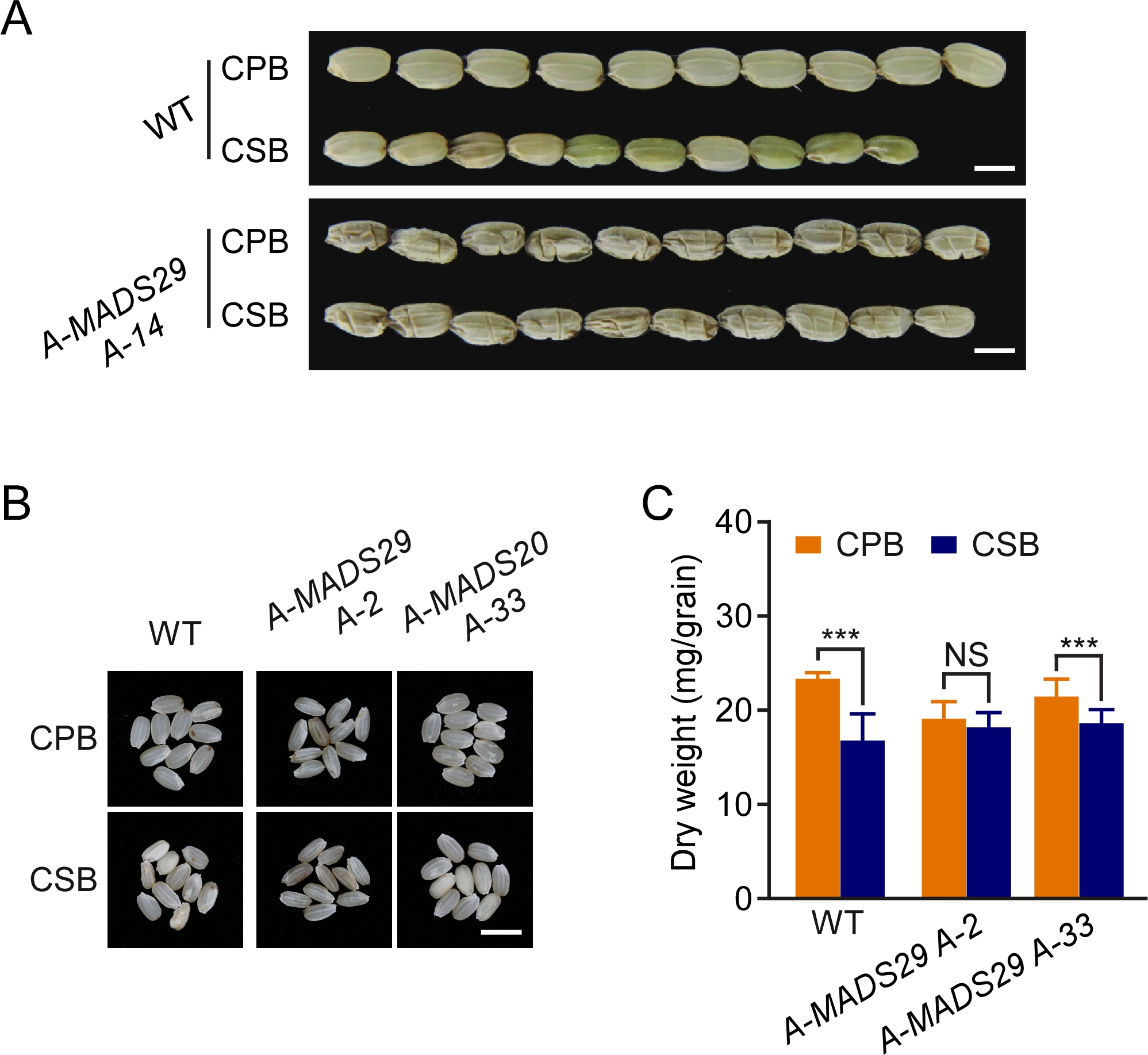

Supplement: S12 Fig — A, Mature grains of CPBs and CSBs in the WT and A-MADS29 A-14 were collected at 40 DAH. Scale bars, 10 mm. B, Developed grains of CPBs and CSBs in the WT, A-MADS29 A-2, and A-33 were collected at 40 DAH. Scale bars, 5 mm. C, Mature grain dry weight of CPBs and CSBs in the WT and A-MADS29 A-2 and A-33 as in (B). Values are means ± SD (n = 30). ***P < 0.001 (Student’s t-test); NS, no significant difference. (JPG) [file pgen.1009157.s016.jpg]
