# Supplementary material for: Auxin apical dominance governed by the OsAsp1-OsTIF1 complex determines distinctive rice caryopses development on different branches
Source: PLoS Genet. 2020 Oct 27;16(10):e1009157. doi: 10.1371/journal.pgen.1009157 (PMC7647119; doi:10.1371/journal.pgen.1009157)
Supplement: Attachment — Submitted filename: Response to reviewers comments.docx [file pgen.1009157.s017.docx]

Response to reviewer’s comments

Reviewer #1:
**Question 1 (Q1)**- P243-244: “The authors back-crossing the T-DNA insertion lines with WT to conclude both asp1-1 and asp1-2 are single-insertion and homozygous-lethal mutants”. The author should provide the test results to support the mutant is a recessive mutation at a single nuclear locus.

**Answer 1 (A1)**: We thank the reviewer’s comments and have shown the genotype results of F1 progeny from two *asp1*^+/–^ independent lines self-crossed or back-crossed with WT and the associated Chi-square test in Supplementary Table 2.

**Q2-** P244-246: “homozygous-lethal mutants, because only about two-third of spikelets developed into fertilized seeds and no homozygous were found in these seeds (Figure 1E and 1F)”. There are two questions. Firstly, two-thirds of spikelets developed into seeds does not match with the Fig1 F, which shows 75% and 72% are fertilized ovary, only one-fourth unfertilized ovary. Secondly, the author should plant more F2 seedlings to do genotyping, to confirm there is no homozygous, other than just calculate the fertilized seeds. It is hard to understand how the author conclude they are homozygous-lethal mutants.

**A2**: We acknowledge the reviewer’s comments and have corrected “two-third of spikelets” to “three quarters of spikelets” in the text because the segregation ratio is 1:2 for a single homozygous-lethal mutant (Page 9, Line 141).

**Q3**- P247-248: “that plant height and spikelet number per panicle were just slightly lower in both asp1-1+/– and asp1-2+/– than in WT”. There is significant difference from the statics results why the author conclude slightly lower. The author should use qRT-PCR to analysis the asp1-1+/– and asp1-2+/– RNA expression levels comparing with the WT, which could possible explain why the two T-DNA insertion lines showing different phenotypes in the following results.

**A3**: We thank the reviewer’s comments and have re-described the results in the text (Page 9, Line 145-148). In addition, we have detected the *OsAsp1* transcription levels in CPB and CSB of WT and *asp1-1*^+/–^ (Figure 1C and 3B). However, the RNA level in *asp1-2^+/-^* is not detected because the following experiments are focused on *asp1-1^+/-^* in this study. From the public transcriptional data of *OsAsp1* in different rice tissues and organs at different development stages on rice genome center (http://rice.plantbiology.msu.edu/expression.shtml), we also knew that *OsAsp1* is highly expressed in spikelets, panicle, endosperm, and stem, but lower in leaves, seedling, and germinated seed. Therefore, we proposed that OsAsp1 is essential for rice growth and caryopsis development.

**Q4**- P290-291: “However, in asp1-1+/–, the expression peaks of OsTAA1 in both CPB and CSB occurred at 3 DAF, which is lower and earlier than in WT”. This conclusion don’t match with the Fig 3C, which show that expression peaks are higher in asp1-1+/– at 3 DAF.

**A4**: we acknowledge the reviewer’s comments and have rephrased the sentence in the text (Page 11, Line 187-189).

**Q5**- P292-294: “the expression pattern of OsYUCCA1 is similar between WT and asp1-1+/– in both CPB and CSB at 5 and 10 DAF, except that OsYUCCA1 expression is lower in CSB of asp1-1+/– as compared to WT (Supplementary Figure 6)”. This conclusion can not match with the figure, because there is no significant difference in CPB of WT and asp1-1+/– at 5 DAF.

**A5**: We thank the reviewer’s comments and have rephrased the sentence in the text (Page 11, Line 189-192).

**Q6**- Fig5D: there are some problems about the Co-IP experiment, causing there is not showing which antibody used as IP from the figures or methods , and there are no anti-HA protein bands. The author should use anti-GFP to do IP, then using anti-HA to detect if there are bands with or without IAA.

**A6**: We acknowledge the reviewer’s comments and have performed the Co-IP experiment to present the update result in the new Figure 5D. In this Co-IP experiment, we had used anti-GFP to detect OsAsp1-GFP and GFP, and anti-HA for OsTIF1-HA, then found that OsTIF1-HA interacts with OsAsp1-GFP, but not GFP.

**Q7**: Since the manuscript conclude the OsAsp1 as a potential target for yield improvement in rice. It is easy to check the yield per plant of asp1-1+/– and asp1-2+/– comparing with the WT.

**A7**: We thank the reviewer’s comments and give the following explanation: Because both of *asp1-1^+/–^* and *asp1-2^+/–^* are single-insertion and homozygous-lethal mutants, one quarter of spikelets of these two lines cannot develop into fertilized seeds. Therefore, there is no necessary for counting their yield. From our results in this study, we conclude that the differential IAA level between CPB and CSB determined by the OsAsp1-OsTIF1 complex is essential for the distinctive development of CPB and CSB, which provides potential targets for engineering yield improvement in crops.

**Q8**: There are over-expression liens of OsAsp1 in the manuscript used in Fig3. Do the authors check the caryopsis phenotypes in the OE-OsAsp1 lines? These results should be more convince for the conclusion from the manuscript.

**A8**: We acknowledge the reviewer’s comments. In Figure 3D and 3E, we heterologously over-expressed *OsAsp1* in *DR5::GUS* transgenic *Arabidopsis thaliana* plants and demonstrated that OsAsp1 upregulates IAA level in *Arabidopsis*. And we have not generated the transgenic rice over-expressed OsAsp1 in this manuscript.

Reviewer #2:

Q9: Authors suggested that high levels of auxin in CPB i.e. auxin apical dominance is key to better grain filling and grain size on CPB compared to CSB. asp mutants showed remarkably low levels or no auxin in both CPB and CSB at different days. The mutant still showed similar (in CPB) or better (in CSB) embryo and endosperm development. How can this be explained if auxin is the key to the studied process?

A9: We acknowledge the reviewer’s comments and have supplemented the discuss contents about auxin roles on rice caryopses development in discuss section (Page 19-20, Line 338-356).

**Q10**: Further, *asp* mutant showed phenotypic effects only on CSB, and no effects on CPB (for grain weight, ovary development and size, endosperm cellularization, and endosperm development). This needs to be explained and justified.

**A10**: We thank the reviewer’s comments and have given the following explanations: *asp1* mutant is homozygous-lethal mutant, which causes about one quarter of spikelets on both primary branch and secondary branch of *asp1^+/-^* developed abortive seeds. Moreover, compared to WT, the difference in the grain weight of developed seeds between CPB and CSB disappeared in both *asp1-1*^+/–^ and *asp1*-*2*^+/–^, and the seed grain weights in *asp1-1*^+/–^ and *asp1*-*2*^+/–^ were also same or closer to CPB in WT. In addition, we rephrased the sentences in discuss section (Page 18, Line 318-322).

**Q11:** What could be the mechanism to achieve initial high levels of IAA that induces OsAsp1 via MADS29? Doesn’t the initial high levels of IAA to induce OsAsp1 require binding of TIF by Asp1? Can it be explained by temporal regulation, or pollination induced IAA level in not dependent upon MADS20, OsAsp1, OsTIF1 and OsTAA1? The initial high levels of IAA, IAA-induced levels of TAA1 to have high IAA levels, and IAA-mediated feedback regulation of the model could possibly be explained more systematically, possibly taking into account temporal and spatial regulation.

**A11:** We acknowledge agree with the reviewer’s comments and have given the following explanations: The various auxin levels in plant cells and tissues depends on both auxin metabolism (biosynthesis, conjugation and degradation) and cellular auxin transport. Figueiredo *et al* (Nature Plants 2015, 1(12): 1-6) showed that auxin is produced post fertilization in seeds, which drives the central cell division and is necessary for MADS-box transcription factor AGL62-dependent endosperm development in *Arabidopsis*. Here, we showed that auxin upregulates *OsAsp1* transcription via MADS29 after fertilization to produce a higher level of *OsAsp1* in CPB than in CSB. Furthermore, the interaction of OsAsp1-OsTIF1 regulated by auxin formed a feedback regulation loop to maintain auxin apical dominance during rice caryopsis development. From our study, we also found the free IAA content significantly higher in CPB than in CSB at the day of fertilization, which is according with previous study (Xu *et al*, Field Crops Research 2007, 101: 53-61). These results indicated that pollination induces higher level of IAA in CPB than in CSB via an uncharacterized mechanism. We totally agree with the reviewer’s concerns about finely temporal and spatial regulation of auxin levels in plants, because there is so many questions need to be dissolved in the future, such as the crosstalk of position information and auxin level between CPB and CSB.

Q12: What could be the reason of very high expression levels of CycB1 in CSB at 3DAF (Figure 2D)? And even at 5DAF, CSB had comparable expression of CycB1 to CPB? The expression levels of TAA1 in WT and mutant is also not fitting well with IAA content (Figure 3 A,B).

A12: We thank the reviewer’s comments and have given the following explanations: Guo *et al* (2010, Planta 231: 293–303) showed that knockdown of *OsCycB1* results in a delayed and abnormal cellularization occurred in the endosperm of rice. Therefore, we used *OsCycB1* as well as OsKRP1 and OsCCS52A as the three molecular markers for the development status of CPB and CSB in WT and *asp1-1^+/–^*. In this study, we just detected the expression of these three genes in CPB and CSB of WT and *asp1-1^+/–^* at 3 and 5 DAF, and found that their expressions are exactly associated with OsAsp1 between CPB and CSB. In Figure 2D, we found higher expression levels of *OsCycB1* in CSB than that in CPB at 3 DAF and the comparable expression in CSB and CPB at 5 DAF, which indicated that the expression peak of *OsCycB1* in CPB of WT reaches earlier than 3 DAF. However, this issue is not the key point for our study.

In addition, we demonstrated that the expression of *OsTAA1*, but not *OsYUCAA1*, is associated with IAA contents in CPB and CSB, which is regulated by OsAsp1-OsTIF1 complex. However, we can not exclude other factors affecting the expression of *OsTAA1* in rice.

**Q13:** The mechanism of Asp1 and TIF function doesn't explain the increased expression levels of TIF1 and TIF2 in asp1 mutant background (Figure 4B).

**A13:** We acknowledge the reviewer’s comments and have discussed the expression of OsTIF1 regulated by OsAsp1 in discuss section (Page 18, Line 327-329): In this study, we found that both disruption of *OsAsp1* level in *asp1-1^+/-^* and overexpression of *OsAsp1* in rice calli activates the transcription of *OsTIF1* (Figure 4B and Figure 4G), indicating that the expression of *OsTIF1* is regulated by a uncharacterized and sophisticated mechanism.

**Q14:** The usage of two different auxins IAA and 2,4 D for different experiments, and different rice genotypes for different experiments shall be justified.

**A14:** We thank the reviewer’s concerns and have given the following explanations: In our lab, we generally planted Zhong Hua 15 annually for rice study. We purchased two independent *asp1* mutant lines from <http://signal.salk.edu>, which have the genetic background of Dong Jin species. In addition, the genetic background of *A-MADS29* transgenic lines is Zhong Hua 11. Therefore, we separately used these three different wild type plants as the control for comparation with their corresponding mutant lines in this study. In addition, we have supplemented the reference for the *in vitro* culture of spikelets treated with 2,4-D and IAA treatment of spikelets on panicle, respectively.
