# Supplementary material for: Auxin apical dominance governed by the OsAsp1-OsTIF1 complex determines distinctive rice caryopses development on different branches
Source: PLoS Genet. 2020 Oct 27;16(10):e1009157. doi: 10.1371/journal.pgen.1009157 (PMC7647119; doi:10.1371/journal.pgen.1009157)
Supplement: Attachment — Submitted filename: Responses to Reviewers Comments.docx [file pgen.1009157.s018.docx]

Response to reviewer’s comments

Reviewer #1:

**Question 1 (Q1): The are two T-DNA insertion lines used in the manuscript, which display different phenotypes in the Sup Fig3. The author did not detect the RNA levels in asp1-1+/– and asp1-2+/ , and the explain is that “the RNA level in asp1-2+/- is not detected because the following experiments are focused on asp1-1+/- in this study.” Why you focused on asp1-1+/- in this study? Since you did not know the RNA levels in the asp1-1+/ and asp1-2+/ comparing with WT.**

**Answer 1 (A1)**: We thank the reviewer’s concerns and have performed the qRT-PCR to monitor the RNA levels in CPB and CSB of *asp1-1^+/-^* and *asp1-2^+/-^* at 0, 3, 5 and 10 DAF. The results showed that the expression of *OsAsp1* in both CPB and CSB of *asp1-1*^+/–^ and *asp1-2*^+/–^ was low and stable, and the expression value at different DAF was similar as that at 0 DAF in CPB and CSB of WT (Figure 1F), indicating that T-DNA insertion decreases the expression of *OsAsp1* in CPB and CSB of two independent lines *asp1-1*^+/–^ and *asp1-2*^+/–^. Therefore, we focused on *asp1-1^+/-^* for the following study.

**Q2: P186-189, Why the OsTAA1 expression peaks occurred at 3 DAF in asp1-1+/- but at 5 DAF in WT? And why higher peak in CPB at WT, but inverse in asp1-1+/- which display higher in CSB? The author should give some explain rather than just rephrased the sentence in the manuscript.**

**A2:** We acknowledge the reviewer’s comments and have given the detail discussion about the transcriptional regulation of *OsTAA1* in discussion section (Page 19, Line 347-359): In WT, the expression of *OsTAA1* was gradually increases after fertilization and reached a peak at 5 DAF in both CPB and CSB of WT, and with a higher peak value in CPB, which was correlated with IAA content between CPB and CSB of WT. However, in *asp1-1*^+/–^, the expression peaks of *OsTAA1* disappeared at 5 DAF, but occurred at 3 DAF in both CPB and CSB, indicating that the transcription of *OsTAA1* is regulated by OsAsp1. In addition, higher expressional peak of OsTAA1 was displayed in CPB at WT, but inverse in *asp1-1^+/-^* which displayed higher in CSB, indicating that there exist some uncharacterized mechanisms on regulating the expression of *OsTAA1* in WT and *asp1-1^+/-^*. However, this issue is not the main point in our manuscript.

**Q3: P189-192, The author should give a little bit explain why the OsYUCCA1 expression pattern display different phenotype in the WT and asp1-1+/-, not just reorganize the sentence.**

**A3:** We thank the reviewer’s comments and also give the detail discussion about the transcriptional regulation of *OsYUCCA1* in discussion section (Page 19, Line 359-365): The expression pattern of *OsYUCCA1*, which was higher expressed in CPB than in CSB at 5 and 10 DAF, respectively, was similar in WT and *asp1-1*^+/–^, except that *OsYUCCA1* expression level was lower in CPB of *asp1-1*^+/–^ than in WT at 10 DAF. These results suggested that OsYUCCA1 is not a main enzyme for IAA biosynthesis between CPB and CSB. Therefore, we will detect the expression levels of other 13 *OsYUCCAs* in CPB and CSB of *asp1-1*^+/–^ in the future study to confirm which *OsYUCCA* is regulated by OsAsp1 and associated with IAA biosynthesis in CPB and CSB.

Reviewer #2:

**Q4: In response to my comment regarding asp mutants showing similar (in CPB) or better (in CSB) embryo and endosperm development despite remarkably low levels or no auxin in both CPB and CSB, authors suggest that low level of auxin is sufficient to trigger embryo and endosperm development. Since this statement is a key to the basic concept of the manuscript, experimental justification of the statement or logical explanation well supported with available literature shall be provided. I still wonder if that very low levels of IAA in asp1 mutant can mediate similar or better cell proliferation than control having very high auxin levels.**

**A4:** We acknowledge the reviewer’s comments and have provided a more reasonable explanations in the discussion section: In this study, we found that *asp1-1^+/-^* presents low content and no distributional difference of auxin in CPB and CSB, which attenuated the difference of ovary development and size, endosperm cellularization and development, and grain weight of developed seeds between CPB and CSB of *asp1-1*^+/–^ and produced heavy grain of CPB and CSB, closer to CPB in WT. Therefore, we concluded that low level of auxin is sufficient to trigger embryo and endosperm development in CPB and CSB, and promotes the development of CSB near to CPB in *asp1-1*^+/–^.

**Q5: Further, authors also argue that it is the difference in the auxin levels, and not the absolute concentration, is critical for maintaining the development difference of CPB and CSB. There should be some more gravity and support to the statement. Is there any threshold to the relative difference in the auxin levels or any minor difference would also work? This is particularly important for asp mutant which has extremely low auxin levels. Further, exogenous auxin treatment of CSB, leading to disappearance of grain weight differences between CPB and CSB, shall be confirmed for the disappearance of the difference in the auxin levels.**

**A5:** We thank the reviewer’s comments and further perform the more experiment to confirm the role of auxin on the developmental difference between CPB and CSB: In this study, we showed that the treatment of CSB in WT with extra IAA before pollination promotes the development of CSB and the grain weight of these CSB rises to that of CPB. Moreover, the treatment of CPB in *asp1-1*^+/–^ with IAA also phenocopied the expression difference of *OsAsp1* and development difference between CPB and CSB of WT. These results suggested that the higher level of auxin in CPB than in CSB, but not absolute IAA concentration, is critical for maintaining the development difference of CPB and CSB.

**Q6: In response to my comment regarding asp mutant showed phenotypic effects only on CSB, and no effects on CPB, the authors explain the grain weight differences in CPB and CSB of mutants.** **However, the question was more towards figure 2 A and B, where wild type and mutant CPB appear very similar for ovary development and size, endosperm cellularization, and endosperm development. This needs to be explained that why asp mutants effect ovary and endosperm development specifically on CSB, and not on CPB. Figure 1G also shows that no significant difference between CPB of wild type and asp1-1. There should be some explanation of why the phenotypic effects is specific to CSB, and not CPB.**

**A6:** We acknowledge the reviewer’s comments and have given the detail explanations in the discussion section: Firstly, we found that both two T-DNA insertion mutant lines *asp1-1* and *asp1-2* are homozygous-lethal, indicating that OsAsp1 is essential for rice caryopses development. Secondly, compared to WT, the difference in the grain weight of developed seeds between CPB and CSB disappeared in both lines, and mature grain weight of CPB and CSB in *asp1-1^+/-^* and *asp1-2^+/-^* are closer to that of CPB in WT, which is caused by disappearance of auxin distribution difference between CPB and CSB and low content of IAA enough for promoting the development of CPB and CSB in the mutant line.

**Q7: Considering the complexity of the process, I think some logical discussion about possibility of temporal and spatial regulation between CPB and CSB may help the manuscript.**

**A7:** We appreciate the reviewer’s comments and have reorganized the discussion section in this manuscript.

**Q8: Regarding my comment on discrepancy in expression levels CycB1, I understand that the expression of cell-cycle genes is not the key point of the study. However, the expression peak of cell-cycle genes in CPB shall overlap with auxin peak and Asp1 expression peak in CPB to have more confidence in the model, as auxin peak has been suggested to be a key to differential embryo and endosperm development via cell proliferation.**

**A8:** We totally agree with the reviewer’s comments and give the following explanations: *OsCycB1*, same as OsKRP1 and OsCCS52A, are proved by three independent research groups as the marker genes associated with cell cycle and division in rice endosperm. However, the expression patterns of these three genes between CPB and CSB were uncharacterized. In this study, we showed that these three genes display differential expression patterns between CPB and CSB in WT and *asp1-1^+/–^* at both 3 and 5 DAF (Figure 2D), indicating the expression of these genes in CPB and CSB are regulated by OsAsp1 during caryopsis development.

**Q9: I still wonder for a reason to use two different auxins: 2,4D for in vitro culture of spikelets, and IAA for treatment of spikelets on panicle.**

**A9:** We acknowledge the reviewer’s comments and have performed the experiment of in vitro culture of spikelets with IAA. Then, we found that IAA induces the expression of OsAsp1 in this experiment (Figure 6A), which is similar as the results of the treatment of 2,4-D for in vitro culture of spikelets (Supplementary Figure 8).

**Q10: The manuscript shall also be thoroughly checked for language and sentence structure.**

**A10:** We thank the reviewer’s suggestion and have requested at least two professional editors, both native speakers of English, to check the English in this manuscript. For a certificate, please see: <http://www.textcheck.com/certificate/IcJQck>.
